# Genomic Prediction for Tuberculosis Resistance in Dairy Cattle

**DOI:** 10.1371/journal.pone.0096728

**Published:** 2014-05-08

**Authors:** Smaragda Tsairidou, John A. Woolliams, Adrian R. Allen, Robin A. Skuce, Stewart H. McBride, David M. Wright, Mairead L. Bermingham, Ricardo Pong-Wong, Oswald Matika, Stanley W. J. McDowell, Elizabeth J. Glass, Stephen C. Bishop

**Affiliations:** 1 The Roslin Institute and RDVS, University of Edinburgh, Midlothian, United Kingdom; 2 Agri-Food and Biosciences Institute, Belfast, United Kingdom; 3 School of Biological Sciences, Queen’s University of Belfast, Belfast, United Kingdom; China Agricultrual University, China

## Abstract

**Background:**

The increasing prevalence of bovine tuberculosis (bTB) in the UK and the limitations of the currently available diagnostic and control methods require the development of complementary approaches to assist in the sustainable control of the disease. One potential approach is the identification of animals that are genetically more resistant to bTB, to enable breeding of animals with enhanced resistance. This paper focuses on prediction of resistance to bTB. We explore estimation of direct genomic estimated breeding values (DGVs) for bTB resistance in UK dairy cattle, using dense SNP chip data, and test these genomic predictions for situations when disease phenotypes are not available on selection candidates.

**Methodology/Principal Findings:**

We estimated DGVs using genomic best linear unbiased prediction methodology, and assessed their predictive accuracies with a cross validation procedure and receiver operator characteristic (ROC) curves. Furthermore, these results were compared with theoretical expectations for prediction accuracy and area-under-the-ROC-curve (AUC). The dataset comprised 1151 Holstein-Friesian cows (bTB cases or controls). All individuals (592 cases and 559 controls) were genotyped for 727,252 loci (Illumina Bead Chip). The estimated observed heritability of bTB resistance was 0.23±0.06 (0.34 on the liability scale) and five-fold cross validation, replicated six times, provided a prediction accuracy of 0.33 (95% C.I.: 0.26, 0.40). ROC curves, and the resulting AUC, gave a probability of 0.58, averaged across six replicates, of correctly classifying cows as diseased or as healthy based on SNP chip genotype alone using these data.

**Conclusions/Significance:**

These results provide a first step in the investigation of the potential feasibility of genomic selection for bTB resistance using SNP data. Specifically, they demonstrate that genomic selection is possible, even in populations with no pedigree data and on animals lacking bTB phenotypes. However, a larger training population will be required to improve prediction accuracies.

## Introduction

Bovine tuberculosis (bTB) is caused by *Mycobacterium bovis*, an aerobic Gram^+^ bacillus and member of the *M. tuberculosis complex*. Cattle (*Bos taurus*) predominantly become infected through the respiratory route and the main lesions observed are tubercles formed in the lungs and draining lymph nodes. BTB is a zoonotic disease and has the potential to impact on animal performance and welfare, causing significant financial losses to the dairy cattle industry worldwide due to production losses and the cost of eradication programmes [1].

Bovine tuberculosis eradication in the UK is impaired by limitations of the available diagnostic and control methods. Diagnosis is based on tuberculin skin testing (Single Intradermal Comparative Cervical test (SICCT) for the UK [2]), post-mortem examination in the abattoir and bacteriological confirmation of infection, all of which suffer imperfect sensitivity. Laboratory confirmation of tuberculin test reactors or suspect abattoir lesions is based on a combination of histology and mycobacterial culture; however, this is complicated by the highly specific requirements of the bacterium *in vitro*. Although the γ-interferon blood test (an alternative diagnostic test) has reportedly higher sensitivity than the standard interpretation SICCT, it has substantially lower specificity. Vaccination using Bacillus Calmette Guerin (BCG) is precluded because vaccinated animals would currently be indistinguishable from infected animals using standard tuberculin tests [3]. Eradication strategies may also be compromised by the presence of a wildlife reservoir, for example the Eurasian badger (*Meles meles)* in the UK and Ireland. Studies on the effectiveness of culling badgers in the UK to reduce bTB prevalence in cattle have shown both positive and negative effects [4].

Following exposure to *M. bovis* only a proportion of animals develop disease, implying variability among individuals in terms of response to infection [5]. Traditional selective breeding requires both phenotypes (i.e. bTB state) and pedigree information. Estimated breeding values (EBVs) can then be calculated using statistical techniques such as best linear unbiased prediction (BLUP). However, such selection would work only on the subset of animals in herds affected by bTB, or their close relatives, and it would require that the population be undergoing an epidemic. Even then, selection intensity would be low if only a small proportion of herds were affected [6]. Therefore, in the case of bTB resistance, it is appealing to be able to identify relatively resistant animals in the absence of phenotypic data from an epidemic.

In contrast to phenotypic selection, genomic selection is a new technology that addresses the problem of identifying relatively resistant individuals by obtaining EBVs for animals without observing phenotypes. Therefore, exposure to infection is not required, at least for several rounds of selection. Genomic selection utilises genomic EBVs estimated directly from SNP data rather than pedigree data (DGVs), calculated as the sum of the effects of genetic markers (Single Nucleotide Polymorphisms, SNPs) across the genome. One method for calculating DGVs is using genomic BLUP (GBLUP) [7], [8]. Through genomic prediction methodology, DGVs may be estimated by combining knowledge on genotypes of the selection candidates and marker effects, and these can then be used as predictors of disease susceptibility for every animal. Genomic prediction in dairy cattle breeding presents certain advantages over phenotypic selection. In particular it improves the rate of genetic gain by shortening the generation intervals, since the DGVs can be calculated as soon as DNA samples are available. Hence, it also allows differentiation between full-sibs, (i.e. prediction of the Mendelian segregation term), without the delay of phenotypic recording [9], [10].

Previous studies have confirmed the presence of potentially exploitable genetic variation in bTB susceptibility among dairy cattle [11], [12]. The hypothesis in the present study is that genetic selection for disease resistance may offer a complementary bTB control strategy, by reducing infection risks and hence contributing to a reduction in herd-level incidence. The aim of this study was to estimate DGVs for bTB resistance by using dense SNP chip data on UK dairy cattle and to test these genomic predictions in the absence of disease phenotype. This is the first step in the investigation of the feasibility of genomic selection for bTB resistance on the basis of predicted DGVs.

## Materials and Methods

### Animals

Phenotypic data for 1,151 cows from 165 dairy cattle herds in Northern Ireland were collected in a case-control study design, with a sample prevalence of 0.51 in the compiled dataset [13]. Information available included bTB skin test data, as described below, the age of the cow on the day of the test, the year when the herd was tested, the season of the test, the reason for which the herd was tested and assigned breed. Animals were tested between August 2008 and September 2009, at a mean age of 4.8 years (ranging from 1 to 11 years); either as part of the annual herd test, herd check tests or reactor herd tests [14]. Most animals were assigned as Holstein adult females, with a small number designated as Friesians (n = 164). A breakdown of data by these variables is given in Table 1.

**Table 1:** The number of animals in the dataset classified by year of test, season of test and reason for test.

	Year		Season			Test reason		
	2008	2009	Winter	Spring	Autumn	Annual	Herd check	Reactor herd
Cases	359	233	309	115	168	155	231	206
Controls	384	175	253	96	210	124	251	184
Totals	743	408	562	211	378	279	482	390

The animal study was licensed by the Department of Health, Social Security and Public Safety for Northern Ireland (DHSSPSNI) under the UK Animals (Scientific Procedures) Act 1986 [ASPA], following a full Ethical Review Process by the Agri-Food & Biosciences Institute (AFBI) Veterinary Sciences Division (VSD) Ethical Review Committee. The study is covered by DHSSPSNI ASPA Project Licence (PPL-2638 ‘Host Genetic Factors in the Increasing Incidence of Bovine Tuberculosis’), and scientists and support staff working with live animals during the studies all hold DHSSPSNI ASPA Personal Licences.

### Phenotype Definitions

Cattle that showed a positive reaction to the Single Intradermal Comparative Cervical test (SICCT), that had TB lesions confirmed by post-mortem examination of carcasses at slaughter and were confirmed as *M. bovis* positive by culture and molecular tests, were defined as cases (592 animals). In this study a positive SICCT was defined as a skin test reaction to *M. bovis* antigens (skin-fold thickness) that, after 72 h exceeds the reaction to *M. avium* antigens by at least 4 mm [2]. Controls were repeatedly SICCT negative and resident >6 months into the episode (559 animals), in herds where cases were observed [13]. Controls were age-matched and preferentially selected from herds with higher disease prevalence in order to increase their probability of exposure to the pathogen [15].

### Genotyping

All individuals were genotyped using BovineHD Illumina Bead Chip. After quality control, 617,885 SNPs were retained for subsequent analysis. Quality control parameters applied included a minimum Gentrain Call (GC) score of 0.60, a minimum minor allele frequency of 0.05 and a minimum call rate of 0.90 for all loci. Animals with a call rate <90% were excluded. The map of the SNP positions was also available (bovine genome assembly *Bos taurus* UMD 3.0).

### Structure Exploration

Principal component analysis (PCA) was used to explore data structure with principal components in *R* (*R version 2.14*). PCA allows discrimination of sample classes and identification of outlier groups representing subpopulations that are genetically distinct. PCA on the 1151×1151 identity-by-state (IBS) matrix of pairwise relatedness, followed by plotting the first principal component values against the second principal component revealed the presence of two clusters, the main one, and a secondary smaller cluster comprising 40 individuals (Figure S1 in [13]) none of which were described as Friesians. By using the BovineHD BeadChip genotypes no sub-structure due to designated animal breed was identified by PCA. Identification of the outliers showed that 39 of them originated from the same herd. Further enquiries revealed that crossbreeding with beef cattle breeds may have taken place in this herd. Thus, to address the possibility of breed differences these animals, along with one additional animal from a different herd that was also clustering with this group, were deleted in some of the following analyses as described in the definition of datasets, below.

### Definition of Datasets

Three slightly different datasets were used in this analysis. Firstly, the full dataset comprising all 1151 individuals was used. Secondly, a reduced dataset was derived from the full dataset, removing the 40 individuals that were identified as outliers by the PCA and for which there was information that they could be crossbreds. This was done in order to address the hypothesis that the presence of beef cross-bred animals may introduce genetic structure to the population and hence alter prediction accuracy. Finally, the analysis was repeated using only animals designated as being Holsteins, after having removed the animals reported by the farmers as Friesians (n = 164). For each analysis and dataset a new **G** matrix was calculated and the corresponding adjusted phenotypes and estimated heritability were obtained (for the analyses excluding the Friesians see details in Supplementary material).

### Calculating Direct Genomic Estimated Breeding Values (DGV)

The aim of the analysis was to estimate the DGVs and then assess their predictive accuracy. To conduct a cross validation analysis, as described below, and to ensure that the sampling of phenotypes would not be biased by the fixed (non-genetic) effects, a two-step approach was used to calculate DGVs. Firstly, the data were pre-corrected for fixed effects, and then random genetic effects, or DGVs, were estimated using the pre-adjusted data [16].

#### 1. Fixed effects model

An initial fixed effects model was used to obtain adjusted phenotypes, corrected for identifiable non-genetic factors. The fixed effects model included animal age, test year, season, test reason and breed as fixed effects, and was fitted using the ASReml package [17]:
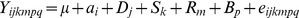
(1)where *Y_ijkmpq_* represents the binary bTB status (0: control, 1: case) of the *q^th^* individual, *µ* is the overall mean, *a_i_* is the age of the individual, *D_j_* is the effect of the year of testing, *S_k_* is the season of testing, *R_m_* is the reason for which testing was initiated in the herd, *B_p_* is the individual’s breed and *e_ijkmpq_* is the residual error. Since all the animals were female and since the controls were selected to originate from herds of higher prevalence, sex and herd of origin were not included in the fixed effects. The residual effects, which are independent of the fixed effects, were obtained and used as phenotypes for the subsequent analyses.

#### 2. Random effects model

The genomic estimated breeding values were calculated for all individuals using the adjusted phenotypes from model (1). As pedigree relationships were unknown in this population, genetic similarities between animals were described using the marker-based IBS genomic kinship (**G**) matrix which has the following elements:
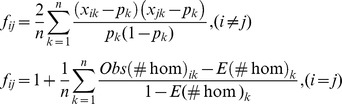
where *x_ik_* (*x_jk_*) is the genotype of the *i^th^* (*j^th^*) animal at the *k^th^* SNP, *n* the total genomic SNPs, and *p_k_* is the frequency of the *B* allele at the *k^th^* SNP. *Obs(#hom)_ik_* and *E(#hom)_k_* are the observed and expected number of homozygous genotypes for the *i^th^* animal at the *k^th^* SNP [18].

To construct **G**, SNPs found only in the homozygote state in the sample and those found on the X chromosome were removed (601,280 SNPs were finally retained in the analysis). From that, the inverse **G** matrix was obtained and used in the random effects model, fitted using the ASReml package, with the following model:
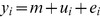
(2)where *m* is the overall mean, ***y*** *_i_* is the residual effect for the *i^th^* individual as calculated from model (1), *u_i_* is it is genomic estimated breeding value with ***u*** ∼ MVN (0, **G**σ_a_^2^) and *e_i_* is its residual value with ***e*** ∼ MVN (0, **I**σ_e_^2^).

#### 3. Full mixed model

For the purpose of estimating the heritability of bTB resistance from the full dataset, the fixed and random effects were fitted simultaneously in ASReml as follows:
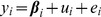
(3)where all the fixed effects (***β***) from model (1) were fitted as before and the relationship information from the **G** matrix was incorporated as random effects.

### Cross Validation

Genomic prediction accuracy can be assessed through cross validation, a non-parametric method that allows assessment of the predictive ability of a classifier [19]. By partitioning the data into a training set and a validation set, DGVs can be predicted for the validation set without reference to phenotypic information. Prediction accuracy can then be calculated by correlating the predicted breeding values and the observed phenotypes, corrected for trait heritability [20]. A five-fold cross validation was conducted as follows.

Firstly, to create the training set in each of the three datasets the individuals were partitioned into five random groups of near-equal size, with the randomization performed separately within the case and control sub-populations. Phenotypes were then masked for each subset in turn, creating five datasets (or folds) in which four-fifths of the animals had a phenotype (training-set, y_1_), and one-fifth had no phenotype (validation-set, y_2_).

Secondly using the GBLUP model (2), predicted DGVs were calculated for each validation-set in turn based on the **G** matrix alone, i.e. information recorded from the training-set animals, (ŷ_2_|y_1_) [20]–[22].

For each of the five test-sets the correlation between the cross-validated predicted DGVs *(ŷ)* and the adjusted phenotypes *(y)*, i.e. *r(y,ŷ),* was calculated. The expected accuracy *(r(g,ĝ))* between the breeding value of an individual *(g)* and its estimate *(ĝ)*, was derived from the correlation as *E[r(g,ĝ)] ≈ r(y, ŷ)/h*, where *h* is the square root of the heritability [20], [23]. The accuracy for each test set was calculated using the heritability obtained for each corresponding cross validation fold and then the average accuracy across all the individuals was obtained.

In order to reduce random sampling effects and assess the sampling properties of the accuracy, the cross validation analysis as described above was replicated six times, where for each replicate a new randomisation was performed so that the individuals comprising each of the groups were different. Finally the average accuracy across all six replications with its empirical 95% confidence interval was obtained, where the confidence interval was calculated from a one sample t-test for the six accuracy values obtained from the six replications.

### Assessing Predictive Ability using ROC Curves

Genomic predictions can be further assessed through the properties of the Receiver Operator Characteristic (ROC) curves and the corresponding area-under-the-ROC-curve (AUC). A ROC curve is the plot of the probability of a positive test result given that the individual is diseased (sensitivity) versus the probability of a positive test result given that the individual is healthy (1-specificity) [24], for all successive thresholds. AUC represents the probability of correct assignment of individuals in the class of diseased or in the class of healthy on the basis of their genotype alone [24]. Using the *R* package, the predicted DGVs for each of the omitted (validation) groups from the cross validation procedure and the binary phenotype for all the 1,151 individuals were used to calculate the ROC curves, along with their corresponding AUC values, for each of the six randomisations for the full dataset.

### Theoretical Expectations

**AUC_max_.** Insight into the information obtained by calculating the ROC curves and their corresponding AUC can be gained by considering these values relative to the theoretical maximum AUC value that could be obtained given the characteristics of the trait and the population under study. Wray et al. (2010) [24] introduced the idea of a maximum AUC value (AUC_max_) that would be achieved if the test classifier was a perfect predictor of genetic risk. This maximum is unique for each disease trait, since it depends on the disease prevalence *(q)* and the heritability of the trait on the underlying liability scale *(h_L_^2^)*. *h_L_^2^* can be estimated from the approximation *h_o_^2^* ∼ *h_L_^2^q^2^i_q_^2^[q(1–q)]^−1^* as introduced by Robertson and Lerner (1949), where *h_o_^2^* is the heritability on the observed scale, *q* is the disease prevalence in the sample and *i_q_* is the mean in standard deviation units of the proportion *q* of the population, assuming a normal distribution. The online calculator provided [24] was used to obtain expected values for AUC_max_ and AUC_half_, which is defined as the AUC expected from a genomic profile that accounts for only a half of the known genetic variance. These values can be used as a basis of comparison for the actual AUC values obtained in the present study.

#### Prediction accuracy

Daetwyler at al. (2010) presented a formula for estimating the expected GBLUP accuracy [8]:
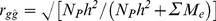
(4)where *N_P_* is the number of individuals in the training population, *h^2^* is the heritability on the observed scale, and *M_e_* is defined as the number of independent chromosome segments which satisfies



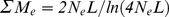
(5)
*M_e_* depends on the genome length in Morgans *L* and on the effective population size *N_e_*. Formulae (4) and (5) were applied to different putative effective population sizes for this sample of animals in order to obtain estimates for the number of independent chromosome segments and the expected corresponding prediction accuracy, for the full dataset and the dataset without the Friesians.

## Results

### GBLUP and Cross Validation

The GBLUP analysis gave an estimate for the heritability of bTB susceptibility of 0.23±0.06 on the observed scale (*h_L_^2^* = 0.34) for the full data set, 0.23±0.07 (*h_L_^2^* = 0.34) for the dataset after removing the 40 individuals identified as a distinct sub-population from the PCA and 0.21±0.07 (*h_L_^2^* = 0.34) for the reduced data set with the Friesian individuals excluded. Table 2 shows the correlations between the adjusted phenotypes and the predicted DGVs, the corresponding heritability estimates and accuracy values with their standard deviations obtained as averages across the five cross validation groups for each of the six replications (detailed tables can be found in the supplementary material, Tables S1, S2, S3 in file S1). Accuracies of 0.33 (95% C.I.: 0.26, 0.40), 0.33 (95% C.I.: 0.28, 0.37), and 0.36 (95% C.I.: 0.33, 0.38) were obtained for the three datasets, respectively. As discussed below, these values are in line with theoretical expectations given the size of the dataset. Further, for each of the cross validation folds and across the six replications, both for the full and the dataset without the Friesians, the observed phenotypes were regressed on the predicted DGVs (Table 3, detailed tables can be found in the Supplementary Material, Tables S4, S5 in file S1). These values are close to the expected value of 1.0.

**Table 2:** Correlations between adjusted phenotypes and predicted DGVs, heritabilities and prediction accuracies.

	Full Dataset			Excluding minor cluster			Excluding Friesians		
	r(ŷ_2_, y_2_)	h^2^	r(g, ĝ) SD	r(ŷ_2_, y_2_)	h^2^	r(g, ĝ) SD	r(ŷ_2_, y_2_)	h^2^	r(g, ĝ) SD
**Run 1**	0.10	0.21	0.22 0.12	0.13	0.21	0.29 0.05	0.13	0.18	0.34 0.22
**Run 2**	0.15	0.19	0.36 0.08	0.15	0.20	0.35 0.10	0.15	0.17	0.38 0.10
**Run 3**	0.15	0.20	0.34 0.14	0.12	0.21	0.29 0.17	0.14	0.18	0.35 0.18
**Run 4**	0.14	0.20	0.33 0.17	0.14	0.20	0.34 0.25	0.15	0.17	0.37 0.16
**Run 5**	0.13	0.20	0.31 0.11	0.16	0.19	0.40 0.21	0.15	0.17	0.37 0.18
**Run 6**	0.17	0.19	0.42 0.18	0.12	0.21	0.28 0.19	0.13	0.18	0.32 0.07
**Average**	0.14	0.20	**0.33 0.07**	0.14	0.21	**0.33 0.05**	0.14	0.18	**0.36 0.02**

r(ŷ_2_, y_2_) is the average correlation between adjusted and predicted phenotypes, h^2^ is the heritability estimate, and r(g,ĝ) is the prediction accuracy with corresponding standard deviation SD. In this table shown are the parameter values for each of the cross validation runs and the averages across all replications for the full data set, the reduced dataset after having removed the animals clustering separately in the PCA, and for the dataset without the animals designated as Friesians.

**Table 3:** Regression of phenotypes on predicted DGVs.

	Regression coefficient	SD	Regression coefficient	SD
**Run 1**	0.74	0.41	1.17	0.87
**Run 2**	1.14	0.27	1.31	0.45
**Run 3**	1.08	0.43	1.22	0.75
**Run 4**	1.16	0.78	1.26	0.55
**Run 5**	1.16	0.78	1.31	0.71
**Run 6**	1.42	0.75	1.06	0.24
**Average**	1.11	0.22	1.22	0.10

Average regression coefficients with the corresponding standard deviations among test sets for each of the cross validation runs and the average across all replications. Left part of the table: full data set, right part: dataset from which the Friesians were excluded.

### ROC Curves and AUC Values

**Figure 1:**
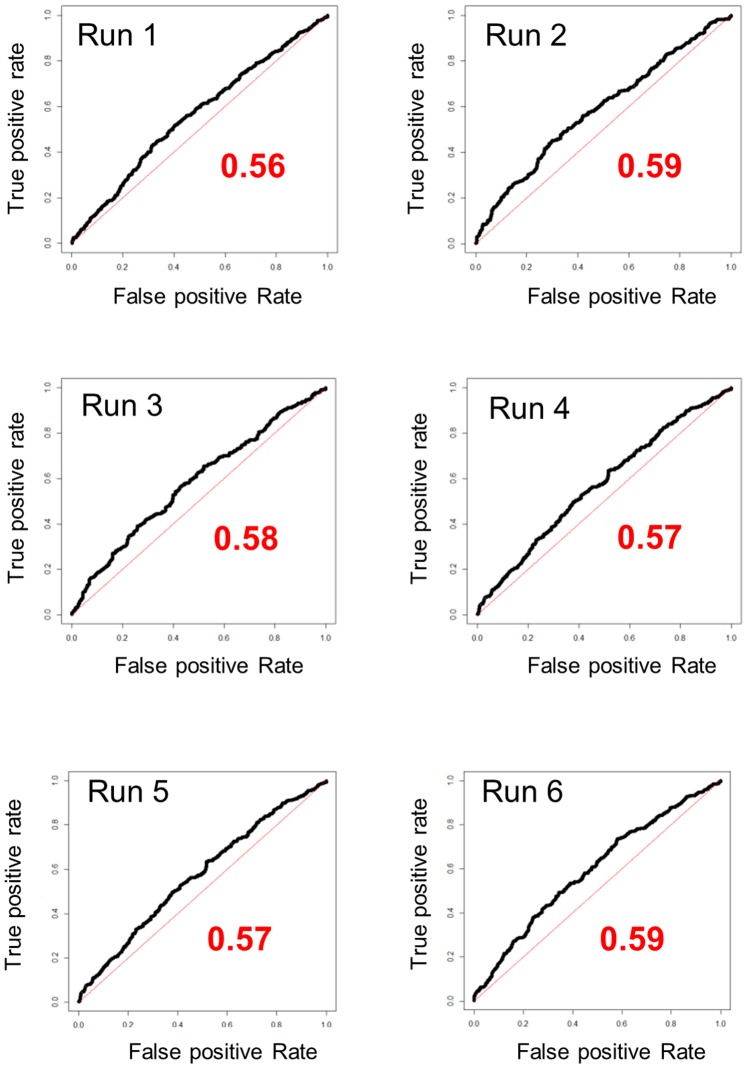
ROC curves for the six randomisations. ROC curves (a plot of true positive rate (Sensitivity) against false positive rate (1-Specificity)) and the corresponding AUC (the probability of correctly assigning an individual as diseased or as healthy on the basis of its genotype alone) for the six randomisation runs for the full dataset.

ROC curves, showing the utility of DGVs as predictors of the binary phenotype, are shown in Figure 1. In these ROC curve plots, the comparison of interest is with the outcome that would be expected by chance (diagonal line of no discrimination). The curves for all randomisations lie above this diagonal line. Therefore, for the population under study the use of genotypes provides information in the prediction of disease state, i.e. the markers help to predict resistance. The AUC values were 0.56, 0.59, 0.58, 0.57, 0.57 and 0.59 for the six different randomizations applied (Figure 1). Hence, there was a probability close to 0.58 of correctly classifying cows based on SNP chip genotype alone using these data. Examples of individual ROC curves for each of the five cross validation test sets within one cross validation run are shown in Figure S1 in file S1.

### Theoretical Expectations

#### AUC values

For the data-set in the present study the disease prevalence was 0.51 (592 cases out of 1,151 animals in total) and *h_L_^2^* was estimated to be 0.34 for a heritability on the observed scale of 0.23. For a prevalence *p* = 0.5, the selection intensity *(i_q_)* would be 0.798 [25]. An AUC_max_ = 0.77 and AUC_half_ = 0.69, can then be obtained using the online calculator provided by Wray et al. (2010). Therefore, the maximum achievable accuracy in this dataset would be 0.77. Our AUC value of 0.58 is somewhat less than AUC_half_, i.e. this is consistent with the accuracy value which also was less than 0.5.

#### Prediction accuracy

Expected accuracies of the genomic predictions are shown in Table 4. With *N_P_* being the average number of individuals in the training population (920.8), *h^2^* the heritability on the observed scale (0.23), the number of independent chromosome segments *M_e_* was calculated for different values of effective population size (Table 4). If Δ*F_g_* is the rate of inbreeding per generation, then for a rate of inbreeding per year Δ*F_y_* = 0.0017 [26] and a five years generation interval for dairy cattle, Δ*F_g_* ≈ 0.01, and thus, a suggestive value for the effective population size would be *N_e_* ≈ 50. Using formulas 4 and 5 with *N_e_* ≈ 50 (M_e_ = 639.79), the expected accuracy would be *r_gĝ_*  = 0.50. Reversing the calculations, an expected accuracy of *r_gĝ_  = *0.34, gives an effective population size of *Ne ≈* 150. This value may not be an unreasonable value for the Holstein-Friesian cows in this sample, given that the population under study is a sample derived as a random selection of non-pedigree dairy cattle and hence possibly not as highly selected as cattle recorded in pedigree databases, and there are likely to be Friesian cows in the dataset along with the possibility of a small number of crossbred animals.

**Table 4:** Expected prediction accuracy for different values of effective population size.

	Full dataset		Excluding Friesians	
	(N_P_ = 920.8 and h^2^ = 0.23)		(N_P_ = 789.6 and h^2^ = 0.21)	
Assumed N_e_	∑M_e_	r_gĝ_	∑M_e_	r_gĝ_
**50**	639.79	0.50	639.79	0.45
**100**	1136.53	0.40	1136.53	0.36
**150**	1600.18	0.34	1600.18	0.31

Training population size (N_p_), heritability, number of independent chromosome segments (∑M_e_) and corresponding expected accuracy (r_gĝ_) for different assumed effective population sizes. Left part of the table: full data set, right part: dataset from which the Friesians were excluded.

For the dataset with the animals designated as Friesians excluded, the expected accuracies were slightly lower, and the observed accuracy was consistent with an effective population size of ca. 100 individuals.

## Discussion

This study provides evidence that genomic selection for bTB resistance is potentially feasible in populations where phenotypic information in unavailable for selection candidates, and even when no pedigree is available. Genomic selection can be considered as a two-step procedure. Initially, on a reference population with both phenotypic and genotypic information, DGVs can be calculated as the genome-wide sum of marker effects [21]. Then, for selection candidates the DGVs can be predicted without the need for phenotypes, since the marker effects have already been calculated in the relevant population [28]. With this design, the results of the present study are important in the context of bTB control. Predicting DGVs in the absence of phenotypes is highly beneficial in the case of bTB, since collection of appropriate phenotypic information requires that a population undergoes an epidemic and that all animals (including controls) are exposed to the pathogen [6]. These conditions can only be met for a subset of animals in the national population and will become increasingly difficult to satisfy as disease prevalence decreases.

The predictive accuracy of the DGVs is at levels that justify further studies on larger populations in order to obtain predictions that could be used in evaluation of selection candidates for their bTB resistance. In order to obtain an accuracy of 0.7, the theoretically required number of animals needed in the training population can be calculated by rearranging formula (4). Given a heritability of 0.23 and with *N_e_ = *50, ∼2,670 individuals would be needed in the training population. But if the *N_e_* were to increase to 100, the size of the required training population would increase to ∼4747 individuals, as might be expected. However, if the *N_e_* was 100 but we targeted a prediction accuracy of 0.5, then the size of the training population needed would reduce to ∼1647. Although in our study, the size of the training population (920.8) was somewhat smaller, the outcomes of the analyses suggest that genomic selection is potentially feasible. However, implementation of genomic selection should wait until we have a greater number of individuals in the training population, to enable us to achieve higher accuracy.

### Estimated Heritability

The data set of UK dairy cattle analysed in this study through the GBLUP approach, provided a heritability estimate of 0.23 (0.34 on the liability scale) for the trait of tuberculosis resistance. This value indicates stronger evidence for genetic variation than previous estimates [11], [12]. However, direct comparison between studies with and without pedigree information should be undertaken with caution. Our estimate is lower than the value reported for deliberately challenged red deer [29]. Health traits often have low heritability due to problems of data collection and interpretation [6]. However, the intermediate heritability of tuberculosis resistance makes genomic selection for tuberculosis resistance an appealing approach to assist in bTB control.

### ROC Curves Properties

A ROC curve is a representation of the different combinations of sensitivity and specificity for successive thresholds between a positive and a negative test result. For a pair of infected and healthy individuals, the probability of correctly identifying the case is represented by the AUC [30]. Although the ROC curves and their AUCs based on genotypic information in this study show only a modest increase in the probability of correctly classifying cases or controls compared to random expectations, these values should also be considered relative to the AUC_max_ [24]. This represents an upper limit of predictive ability given the properties of the dataset and the trait under study, assuming that the classifier (i.e. the DGVs) were a perfect predictor of genetic risk. Since AUC_max_ depends on disease prevalence and trait heritability, the authors argue that prediction accuracy measure should be preferred for genomic prediction evaluation [31], as it is independent of the epidemic properties.

### Cross Validation Prediction Accuracy

Random error due to sampling effects was minimized by averaging the accuracies across several replications with different randomizations so that the individuals comprising each of the five groups were different each time. The differences observed between the randomizations indicate that even with ca. 1000 individuals, random sampling effects still contribute significantly to the cross validation outcomes. Conducting more randomisations was preferred to increasing the number of groups i.e. cross validation folds, because the test set would be reduced, increasing variability across the cross validation folds.

When the full dataset was used, the accuracy obtained was consistent with the theoretical accuracy obtained using the formula by Daetwyler et al. (2008) for an effective population size of *N_e_* = 150, given the properties of the dataset (i.e. sample size and trait heritability). This *N_e_* value is somewhat higher than that often suggested for the Holstein cattle population (c.f. *N_e_* ca. 50 [27]), but may have been inflated due to the structure present in the dataset revealed by PCA, and also from the designation of several individuals as Friesian. Both factors would increase the apparent *N_e_*. Further, the population under study is not a pedigree or a highly selected population, with the animals included in the study sampled from random commercial farms.

It should be noted that results from the different variations of the datasets used were coherent across the analyses. When the cows designated as Friesians were removed, in addition to giving slightly increased accuracy, the dataset behaved more consistently across replicates, and the corresponding implied *N_e_* was reduced (*N_e_* ca. 100). This was despite the fact that the dataset was smaller; presumably reflecting a more uniform population with linkage disequilibrium extending across longer chromosomal regions. Removing the PCA outliers had little impact on the prediction accuracy, however the number removed (14 cases and 26 controls) may have been too few to affect the results.

Phenotypic selection based on EBVs remains a possibility for bTB, but collection of enough phenotypic data to accurately estimate EBVs across an entire population is challenging since it requires the presence of an epidemic. Even if pedigree-recorded herds were affected, providing complete and good quality data, as is the case in the UK, analysing these data would only provide results with an application to specific sub-populations, i.e. animals that are more closely related to the herd. For animals that are more distantly related by pedigree to the ones in the epidemic, accuracy of the pedigree-based EBVs would be poor. Genomic selection for bTB resistance overcomes this problem and thus is potentially very useful, even if prediction accuracy is only modest.

Finally it has been estimated that the basic reproductive number (*R_0_*), i.e. the average number of cases generated by one infectious individual, for bTB in the UK is only slightly greater than 1 (1.07) and so even a modest intervention would be sufficient to substantially reduce the risks or severities of bTB breakdowns [32]. Similarly, even when *R_0_* is substantially greater than one as in the case of the UK foot-and-mouth disease epidemic, a combination of intervention strategies can substantially contribute towards bringing the epidemic under control [33]. Selection to make animals more resistant would help to reduce *R_0_* for bTB.

### Conclusion

Our results demonstrate that genomic selection is potentially feasible for bTB resistance even in populations with no pedigree data available, and it can be applied to animals lacking bTB phenotypes. Potentially this technique could also be applied to other diseases such as Paratuberculosis (Johne’s disease). Access to a greater number of animal phenotypes, thereby creating larger training sets, would help to improve potential prediction accuracies and open up opportunities for implementation.

## Supporting Information

File S1 **File S1 includes the following: Figure S1. ROC curves for each of the five cross validation test groups.** For the full data set, the ROC curves for each of the five cross validation test groups are presented for the first randomisation. **Table S1. Detailed accuracy tables for the six randomisations** **for the data set including all the individuals.** For the data set including all the individuals, the correlation, heritability with its standard error and corresponding prediction accuracy for each of the five test-groups from the Cross Validation procedure are presented for the six different randomization replications. **Table S2. Detailed accuracy tables for the six randomisations for the data set in which animals clustering separately in the PCA were removed.** For the data set in which animals clustering separately in the PCA were removed, the correlation, heritability with its standard error and corresponding prediction accuracy for each of the five test-groups from the Cross Validation procedure are presented for the six different randomisation replications. **Table S3. Detailed accuracy tables for the six randomisations for the data set when the 164 animals designated as Friesians were removed.** The correlation, heritability with standard errors and corresponding prediction accuracy for each of the five test groups in the Cross Validation procedure resulting from the six randomisation replications when the 164 animals designated as Friesians were removed. The data for the remaining 987 animals were re-randomised to training and test sets, which were ∼790 and ∼198 respectively. In the initial fixed effects model breed was removed from the fixed effects and a new **G** matrix calculated only for the Holsteins was used. **Table S4. Detailed results for the regression analysis on the full dataset.** For the full datset, intercept (a) and regression coefficients (b) for the regresison of adjusted phenotypes (observed) on cross-validated EBVs (predicted), for each cross validation fold across the six replication runs, with corresponding standard deviations. **Table S5. Detailed results for the regression analysis on the datset without the Friesians.** For the datset without the Friesians, intercept (a) and regression coefficients (b) for the regression of adjusted phenotypes (observed) on cross validated EBVs (predicted), for each cross validation fold across the six replication runs, with corresponding standard deviations.
(DOCX)
