# Supplementary material for: Genomic Prediction for Tuberculosis Resistance in Dairy Cattle
Source: PLoS One. 2014 May 8;9(5):e96728. doi: 10.1371/journal.pone.0096728 (PMC4014548; doi:10.1371/journal.pone.0096728)
Supplement: File S1 — includes the following: Figure S1. ROC curves for each of the five cross validation test groups. For the full data set, the ROC curves for each of the five cross validation test groups are presented for the first randomisation. Table S1. Detailed accuracy tables for the six randomisations for the data set including all the individuals. For the data set including all the individuals, the correlation, heritability with its standard error and corresponding prediction accuracy for each of the five test-groups from the Cross Validation procedure are presented for the six different randomization replications. Table S2. Detailed accuracy tables for the six randomisations for the data set in which animals clustering separately in the PCA were removed. For the data set in which animals clustering separately in the PCA were removed, the correlation, heritability with its standard error and corresponding prediction accuracy for each of the five test-groups from the Cross Validation procedure are presented for the six different randomisation replications. Table S3. Detailed accuracy tables for the six randomisations for the data set when the 164 animals designated as Friesians were removed. The correlation, heritability with standard errors and corresponding prediction accuracy for each of the five test groups in the Cross Validation procedure resulting from the six randomisation replications when the 164 animals designated as Friesians were removed. The data for the remaining 987 animals were re-randomised to training and test sets, which were ∼790 and ∼198 respectively. In the initial fixed effects model breed was removed from the fixed effects and a new G matrix calculated only for the Holsteins was used. Table S4. Detailed results for the regression analysis on the full dataset. For the full datset, intercept (a) and regression coefficients (b) for the regresison of adjusted phenotypes (observed) on cross-validated EBVs (predicted), for each cross validation fold ac [file pone.0096728.s001.docx]

**Supporting Information**

**Figure S1. ROC curves for each of the five cross validation test groups.**

**
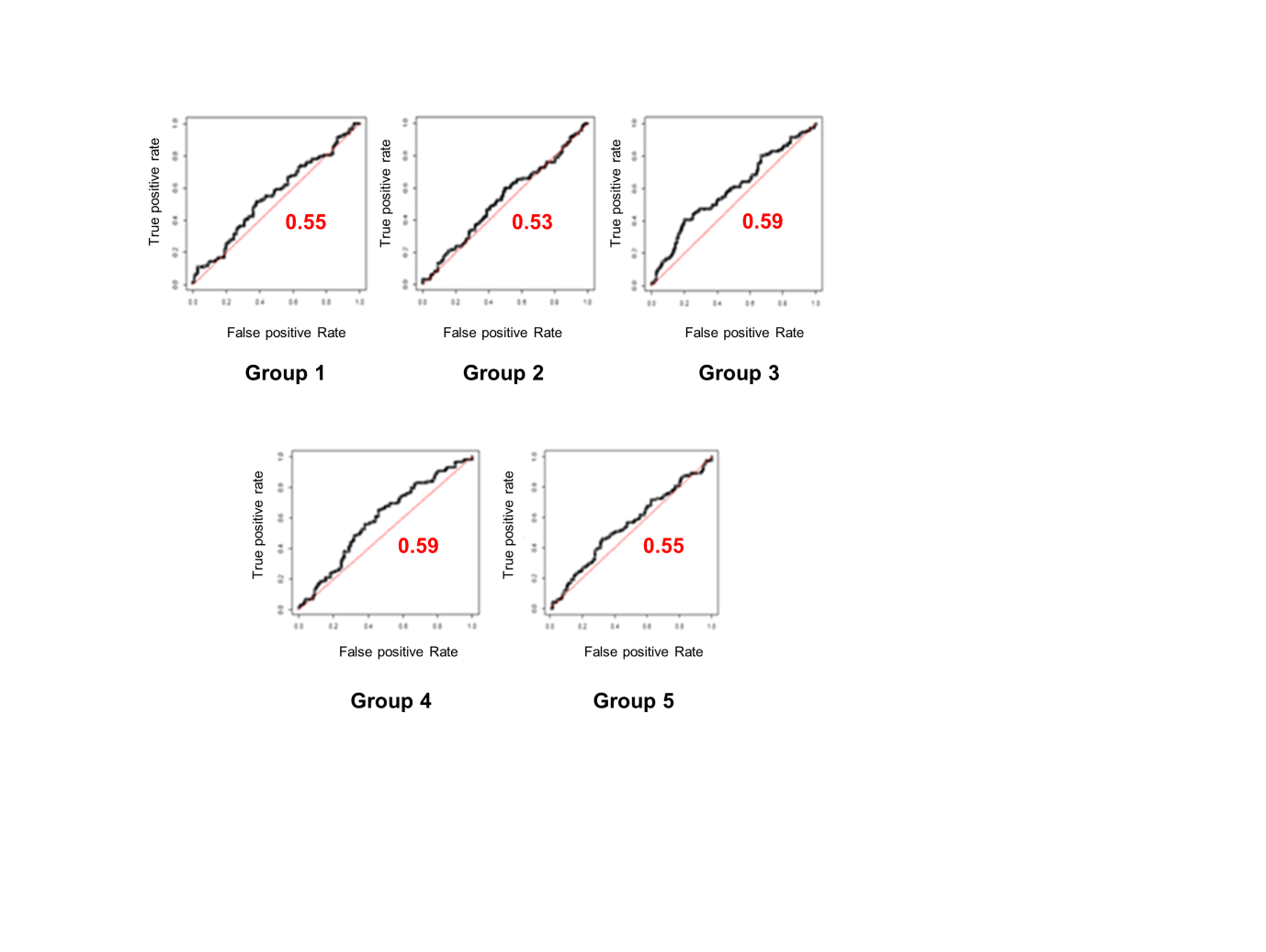
**

For the full data set, the ROC curves for each of the five cross validation test groups are presented for the first randomisation.

**Table S1. Detailed accuracy tables for the six randomisations** **for the data set including all the individuals.**

|  | **Run 1** | | | | **Run 2** | | | | **Run 3** | | | | **Run 4** | | | | **Run 5** | | | | **Run 6** | | | |
| --- | --- | --- | --- | --- | --- | --- | --- | --- | --- | --- | --- | --- | --- | --- | --- | --- | --- | --- | --- | --- | --- | --- | --- | --- |
|  | **r(ŷ_2_,y_2_)** | **h^2^ and SE** | | **r(g,ĝ)** | **r(ŷ_2_, y_2_)** | **h^2^ and SE** | | **r(g,ĝ)** | **r(ŷ_2_,y_2_)** | **h^2^ and SE** | | **r(g,ĝ)** | **r(ŷ_2_,y_2_)** | **h^2^ and SE** | | **r(g,ĝ)** | **r(ŷ_2_,y_2_)** | **h^2^ and SE** | | **r(g,ĝ)** | **r(ŷ_2_,y_2_)** | **h^2^ and SE** | | **r(g,ĝ)** |
| **group 1** | 0.08 | 0.23 | 0.08 | 0.17 | 0.13 | 0.20 | 0.08 | 0.30 | 0.20 | 0.16 | 0.08 | 0.50 | 0.11 | 0.22 | 0.08 | 0.24 | 0.07 | 0.22 | 0.08 | 0.15 | 0.19 | 0.18 | 0.08 | 0.45 |
| **group 2** | 0.08 | 0.22 | 0.08 | 0.16 | 0.17 | 0.19 | 0.08 | 0.40 | 0.08 | 0.20 | 0.07 | 0.17 | 0.12 | 0.25 | 0.08 | 0.24 | 0.20 | 0.25 | 0.08 | 0.40 | 0.11 | 0.23 | 0.08 | 0.23 |
| **group 3** | 0.15 | 0.17 | 0.07 | 0.33 | 0.18 | 0.17 | 0.08 | 0.44 | 0.12 | 0.25 | 0.08 | 0.25 | 0.22 | 0.13 | 0.07 | 0.62 | 0.14 | 0.13 | 0.07 | 0.39 | 0.24 | 0.12 | 0.07 | 0.70 |
| **group 4** | 0.16 | 0.18 | 0.07 | 0.36 | 0.12 | 0.22 | 0.08 | 0.24 | 0.18 | 0.17 | 0.08 | 0.44 | 0.10 | 0.19 | 0.07 | 0.23 | 0.10 | 0.19 | 0.07 | 0.23 | 0.13 | 0.21 | 0.08 | 0.29 |
| **group 5** | 0.05 | 0.25 | 0.08 | 0.09 | 0.17 | 0.17 | 0.07 | 0.41 | 0.16 | 0.20 | 0.08 | 0.36 | 0.15 | 0.19 | 0.07 | 0.33 | 0.16 | 0.19 | 0.07 | 0.36 | 0.18 | 0.20 | 0.08 | 0.41 |
| **average** | 0.10 | 0.21 |  | **0.22** | 0.15 | 0.19 |  | **0.36** | 0.15 | 0.20 |  | **0.34** | 0.14 | 0.20 |  | **0.33** | 0.13 | 0.20 |  | **0.31** | 0.17 | 0.19 |  | **0.42** |

For the data set including all the individuals, the correlation, heritability with its standard error and corresponding prediction accuracy for each of the five test-groups from the Cross Validation procedure are presented for the six different randomization replications.

**Table S2. Detailed accuracy tables for the six randomisations** **for the data set in which animals clustering separately in the PCA were removed.**

|  | **Run 1** | | | | **Run 2** | | | | **Run 3** | | | | **Run 4** | | | | **Run 5** | | | | **Run 6** | | | |
| --- | --- | --- | --- | --- | --- | --- | --- | --- | --- | --- | --- | --- | --- | --- | --- | --- | --- | --- | --- | --- | --- | --- | --- | --- |
|  | **r(ŷ_2_, y_2_)** | **h^2^ and SE** | | **r(g, ĝ)** | **r(ŷ_2_, y_2_)** | **h^2^ and SE** | | **r(g, ĝ)** | **r(ŷ_2_, y_2_)** | **h^2^ and SE** | | **r(g, ĝ)** | **r(ŷ_2_, y_2_)** | **h^2^ and SE** | | **r(g, ĝ)** | **r(ŷ_2_, y_2_)** | **h^2^ and SE** | | **r(g, ĝ)** | **r(ŷ_2_, y_2_)** | **h^2^ and SE** | | **r(g, ĝ)** |
| **group 1** | 0.15 | 0.18 | 0.08 | 0.36 | 0.13 | 0.26 | 0.09 | 0.25 | 0.06 | 0.27 | 0.08 | 0.12 | 0.05 | 0.25 | 0.08 | 0.10 | 0.17 | 0.22 | 0.08 | 0.36 | 0.08 | 0.25 | 0.08 | 0.15 |
| **group 2** | 0.13 | 0.20 | 0.08 | 0.29 | 0.18 | 0.15 | 0.08 | 0.45 | 0.13 | 0.17 | 0.08 | 0.31 | 0.20 | 0.15 | 0.08 | 0.50 | 0.12 | 0.22 | 0.08 | 0.26 | 0.15 | 0.23 | 0.08 | 0.32 |
| **group 3** | 0.14 | 0.20 | 0.08 | 0.31 | 0.20 | 0.18 | 0.08 | 0.46 | 0.17 | 0.18 | 0.08 | 0.40 | 0.25 | 0.13 | 0.08 | 0.69 | 0.23 | 0.14 | 0.08 | 0.62 | 0.03 | 0.26 | 0.08 | 0.06 |
| **group 4** | 0.12 | 0.22 | 0.08 | 0.26 | 0.15 | 0.21 | 0.08 | 0.32 | 0.19 | 0.15 | 0.08 | 0.50 | 0.14 | 0.20 | 0.08 | 0.31 | 0.08 | 0.28 | 0.09 | 0.15 | 0.14 | 0.19 | 0.08 | 0.32 |
| **group 5** | 0.11 | 0.23 | 0.08 | 0.24 | 0.13 | 0.21 | 0.08 | 0.28 | 0.06 | 0.30 | 0.09 | 0.10 | 0.06 | 0.29 | 0.09 | 0.11 | 0.21 | 0.11 | 0.07 | 0.62 | 0.21 | 0.14 | 0.08 | 0.56 |
| **average** | 0.13 | 0.21 |  | **0.29** | 0.15 | 0.20 |  | **0.35** | 0.12 | 0.21 |  | **0.29** | 0.14 | 0.20 |  | **0.34** | 0.16 | 0.19 |  | **0.40** | 0.12 | 0.21 |  | **0.28** |

For the data set in which animals clustering separately in the PCA were removed, the correlation, heritability with its standard error and corresponding prediction accuracy for each of the five test-groups from the Cross Validation procedure are presented for the six different randomisation replications.

**Table S3. Detailed accuracy tables for the six randomisations for the data set when the 164 animals designated as Friesians were removed.**

|  | **Run 1** | | | | **Run 2** | | | | **Run 3** | | | | **Run 4** | | | | **Run 5** | | | | **Run 6** | | | |
| --- | --- | --- | --- | --- | --- | --- | --- | --- | --- | --- | --- | --- | --- | --- | --- | --- | --- | --- | --- | --- | --- | --- | --- | --- |
|  | **r(ŷ_2_,y_2_)** | **h^2^ and SE** | | **r(g,ĝ)** | **r(ŷ_2_ y_2_)** | **h^2^ and SE** | | **r(g,ĝ)** | **r(ŷ_2_,y_2_)** | **h^2^ and SE** | | **r(g,ĝ)** | **r(ŷ_2_ y_2_)** | **h^2^ and SE** | | **r(g, ĝ)** | **r(ŷ_2_,y_2_)** | **h^2^ and SE** | | **r(g,ĝ)** | **r(ŷ_2_,y_2_)** | **h^2^ and SE** | | **r(g,ĝ)** |
| **group 1** | 0.17 | 0.14 | 0.08 | 0.44 | 0.18 | 0.16 | 0.08 | 0.45 | 0.17 | 0.13 | 0.07 | 0.47 | 0.15 | 0.19 | 0.08 | 0.33 | 0.04 | 0.26 | 0.09 | 0.08 | 0.15 | 0.16 | 0.08 | 0.38 |
| **group 2** | 0.23 | 0.13 | 0.08 | 0.63 | 0.18 | 0.13 | 0.07 | 0.51 | 0.15 | 0.19 | 0.08 | 0.34 | 0.07 | 0.20 | 0.08 | 0.17 | 0.13 | 0.17 | 0.08 | 0.31 | 0.14 | 0.16 | 0.08 | 0.35 |
| **group 3** | 0.09 | 0.18 | 0.08 | 0.21 | 0.15 | 0.18 | 0.08 | 0.36 | 0.13 | 0.22 | 0.09 | 0.28 | 0.17 | 0.14 | 0.08 | 0.47 | 0.18 | 0.14 | 0.08 | 0.49 | 0.12 | 0.17 | 0.08 | 0.30 |
| **group 4** | 0.15 | 0.18 | 0.08 | 0.36 | 0.14 | 0.16 | 0.08 | 0.33 | 0.05 | 0.26 | 0.09 | 0.09 | 0.14 | 0.19 | 0.09 | 0.32 | 0.20 | 0.15 | 0.08 | 0.52 | 0.16 | 0.19 | 0.09 | 0.38 |
| **group 5** | 0.02 | 0.25 | 0.09 | 0.04 | 0.12 | 0.23 | 0.09 | 0.25 | 0.19 | 0.12 | 0.08 | 0.57 | 0.21 | 0.13 | 0.08 | 0.58 | 0.17 | 0.16 | 0.08 | 0.44 | 0.10 | 0.22 | 0.09 | 0.21 |
| **average** | 0.13 | 0.18 |  | **0.34** | 0.15 | 0.17 |  | **0.38** | 0.14 | 0.18 |  | **0.35** | 0.15 | 0.17 |  | **0.37** | 0.15 | 0.17 |  | **0.37** | 0.13 | 0.18 |  | **0.32** |

The correlation, heritability with standard errors and corresponding prediction accuracy for each of the five test groups in the Cross Validation procedure resulting from the six randomisation replications when the 164 animals designated as Friesians were removed. The data for the remaining 987 animals were re-randomised to training and test sets, which were ~790 and ~198 respectively. In the initial fixed effects model breed was removed from the fixed effects and a new **G** matrix calculated only for the Holsteins was used.

**Table S4. Detailed results for the regression analysis on the full dataset.**

|  | **group 1** | | **group 2** | | **group 3** | | **group 4** | | **group 5** | |  |  |
| --- | --- | --- | --- | --- | --- | --- | --- | --- | --- | --- | --- | --- |
|  | **a** | **b** | **a** | **b** | **a** | **b** | **a** | **b** | **a** | **b** | **intercept SD** | **regr coef SD** |
| **Run 1** | -0.002 | 0.580 | -0.014 | 0.502 | 0.002 | 1.099 | 0.017 | 1.237 | 0.006 | 0.279 | 0.011 | 0.410 |
| **Run 2** | -0.014 | 0.937 | 0.004 | 1.345 | 0.009 | 1.302 | -0.004 | 0.766 | 0.004 | 1.333 | 0.009 | 0.268 |
| **Run 3** | 0.023 | 1.474 | -0.006 | 0.511 | -0.007 | 0.783 | -0.001 | 1.506 | -0.002 | 1.108 | 0.012 | 0.433 |
| **Run 4** | 0.012 | 0.834 | -0.011 | 0.656 | -0.003 | 2.530 | -0.007 | 0.701 | 0.011 | 1.063 | 0.010 | 0.784 |
| **Run 5** | 0.012 | 0.834 | -0.011 | 0.656 | -0.003 | 2.530 | -0.007 | 0.701 | 0.011 | 1.064 | 0.010 | 0.784 |
| **Run 6** | 0.002 | 1.491 | 0.006 | 0.636 | 0.004 | 2.598 | -0.006 | 0.950 | -0.005 | 1.400 | 0.005 | 0.746 |

For the full datset, intercept (a) and regression coefficients (b) for the regresison of adjusted phenotypes (observed) on cross-validated EBVs (predicted), for each cross validation fold across the six replication runs, with corresponding standard deviations.

**Table S5. Detailed results for the regression analysis on the datset without the Friesians.**

|  | **group 1** | | **group 2** | | **group 3** | | **group 4** | | **group 5** | |  |  |
| --- | --- | --- | --- | --- | --- | --- | --- | --- | --- | --- | --- | --- |
|  | **a** | **b** | **a** | **b** | **a** | **b** | **a** | **b** | **a** | **b** | **intercept SD** | **regr coef SD** |
| **Run 1** | 0.001 | 1.371 | 0.005 | 2.429 | 0.005 | 0.635 | -0.006 | 1.325 | 0.002 | 0.114 | 0.004 | 0.873 |
| **Run 2** | 0.000 | 1.629 | -0.008 | 1.918 | 0.007 | 1.185 | -0.001 | 1.001 | -0.002 | 0.834 | 0.005 | 0.450 |
| **Run 3** | -0.001 | 1.747 | 0.010 | 1.093 | 0.007 | 0.860 | -0.001 | 0.261 | -0.017 | 2.157 | 0.011 | 0.745 |
| **Run 4** | 0.002 | 1.077 | 0.004 | 0.542 | 0.000 | 1.754 | -0.009 | 1.055 | 0.010 | 1.870 | 0.007 | 0.549 |
| **Run 5** | -0.002 | 0.228 | 0.002 | 1.095 | -0.008 | 1.769 | 0.005 | 2.071 | 0.007 | 1.385 | 0.006 | 0.709 |
| **Run 6** | -0.006 | 1.229 | 0.005 | 1.200 | -0.006 | 1.043 | 0.000 | 1.176 | 0.010 | 0.651 | 0.007 | 0.240 |

For the datset without the Friesians, intercept (a) and regression coefficients (b) for the regression of adjusted phenotypes (observed) on cross validated EBVs (predicted), for each cross validation fold across the six replication runs, with corresponding standard deviations.
